# Impacts and interactions of organic compounds with chlorine sanitizer in recirculated and reused produce processing water

**DOI:** 10.1371/journal.pone.0208945

**Published:** 2018-12-12

**Authors:** Zi Teng, Sam van Haute, Bin Zhou, Cathleen J. Hapeman, Patricia D. Millner, Qin Wang, Yaguang Luo

**Affiliations:** 1 U. S. Department of Agriculture, Agricultural Research Service, Beltsville Agricultural Research Center, Environmental Microbiology and Food Safety Laboratory, Beltsville, Maryland, United States of America; 2 Department of Nutrition and Food Science, University of Maryland, College Park, Maryland, United States of America; 3 U. S. Department of Agriculture, Hydrology and Remote Sensing Laboratory, Beltsville, Maryland, United States of America; Kyungpook National University, REPUBLIC OF KOREA

## Abstract

Water conservation and economics dictate that fresh produce processors reuse/recirculate the process water. However, the ensuing accumulation of organic matter in water depletes the chlorine sanitizer required for food safety. In this study, we comprehensively investigated chemical compounds that are responsible for water quality in relation to chemical oxygen demand (COD) and chlorine demand (CLD), the two most critical factors associated with water treatment and chlorine replenishment. Simulating commercial fresh-cut wash operations, multiple batches of diced cabbage (0.3 x 0.3 cm2) were washed in the same tank of water. The major components were isolated from the wash water and analyzed by HPLC. Sugars were the predominant compounds (82.7% dry weight) and the major contributor to COD (81.6%), followed by proteins/peptides (7.3% dry weight, 5.3% COD), organic acids (6.2% dry weight, 3.6% COD), and phenolics (0.5% dry weight, 0.5% COD). By repeated time course measures, the effect of these chemicals on CLD are dependent on the chemical structure, concentration in the wash water, and their rate of reaction. Proteins/peptides accounted for about 50% of the total CLD over a 120-min period and phenolics was 21% at 5 min, but diminished with time. The contribution by organic acids and sugars increased continuously, reaching 22% and 16% of total CLD at 120 min of chlorination, respectively. Collectively, these compounds represented 86% of the CLD in cabbage wash water at 5 min and greater than 94% CLD afterwards. This is the first systematic report on the source of COD and CLD during fresh produce washing. It provides essential information for the produce processors to develop safe, effective, and economical wash water treatment/reuse and chlorine replenishment strategies.

## Introduction

Advances in water reuse technology implemented in the fresh produce industry continue to save large quantities of water [1] and associated costs required to remove soil, field debris, and pathogens during fresh-cut processing and other post-harvest handling applications [2, 3]. However, reuse of water results in a high organic load, and subsequently a large chemical oxygen demand (COD), due to the release of naturally-present organic constituents from the cut or damaged plant tissues [4]. These organic constituents provide copious nutrients that support microbial growth, and thus must be removed to meet national and international regulations for water reuse [5, 6]. In addition, these organic constituents generate a high chlorine demand (CLD) leading to rapid depletion of free chlorine (FC) disinfectant in reuse process water and potential for pathogen cross-contamination with significant public health consequences. FC levels can be restored by adding concentrated sodium hypochlorite, but repeated additions to water with high organic load can render it ineffective and result in the formation of toxic chlorine byproducts [7].

Although general information is available regarding the nutritional composition of vegetables, little is known regarding the chemical types, abundance, and reactivity of major organic compounds in fresh produce wash water in relation to COD and CLD. Prior studies evaluated short-term chlorination kinetics (within 1.5 min) of model compounds present in vegetables [8], and chlorine loss due to individual organic compounds relative to pH level [9]. Additional information on these chemical compounds, their prevalence in wash water, and their actual contribution to COD and CLD in longer periods, is needed to determine their impacts and to develop control strategies for improved safe water reconditioning and reuse throughout the high volume fresh-cut produce industry.

Predictive models have been considered for estimating the CLD using protein and phenolic contents, as well as the UV absorbance at 254 nm (UV_254_) as an indicator [10]. However, the degree of success using these models depended on the type of produce. This is partly attributable to the great diversity of phenolics and proteins/peptides, which cannot be characterized satisfactorily by one general parameter or a single model compound. In addition, the correlation between UV_254_ and CLD does not necessarily confirm that phenolics and/or proteins/peptides are the major contributors to CLD. Other potential candidates such as sugars and organic acids were not considered in those models [11]. To date, a comprehensive profile of organics in fresh produce wash water has not been established; therefore, identification of the primary sources of CLD remains elusive.

The major objective of this study was to identify, characterize, and quantify the major classes of organic compounds in wash water and their contributions to COD and CLD. Diced cabbage was chosen as a model product because of its high COD level (up to 8,000 mg/L) observed in industrial washing practices and the extreme challenge reported by the produce industry [4] regarding maintaining FC concentration required to prevent pathogen survival in water.

## Materials and methods

### Materials

White cabbage (*Brassica oleracea* var. *capitate* L.) was purchased from a local wholesale market (Jessup, MD, USA), and stored at 4°C prior to processing. All chemicals and solvents (including water) for chromatographic analyses were of HPLC grade (Sigma Aldrich, St. Louis, MO, USA). All other reagents including acids, bases, model compounds, and sodium hypochlorite, were of analytical grade (Sigma Aldrich). Distilled water was used for all non-HPLC studies.

### Preparation of cabbage exudate and wash water

Cabbage was decored and cut into approximately 0.3 x 0.3 cm^2^ cubes using a Sprint 2 Dicer (Urschel Laboratory, Valparaiso, IN, USA) according to typical industry practice. Six batches of diced cabbage (600 g each) were washed sequentially in 3,000 mL of distilled water with manual agitation. Diced cabbage was discarded and the water collected. Cabbage exudate was prepared from decored cabbage using an Oster Sunbeam household juice extractor (Model 3165, Sunbeam Product Inc., Boca Raton, FL, USA). Wash water and exudate were freeze-dried or frozen immediately, and separately, then stored at -80°C until analyses. The weight of cabbage used for wash water (3,600 g) and exudate (500 g), and the total volume of wash water (3,000 mL) and exudate (450 mL) generated were used to develop a conversion factor (CF) for each class of analyte in the wash water relative to that in the exudate.

$$CF\;\left(\%\right)=\frac{Mass\;of\;analyte\;released\;from\;1\;g\;cabbage\;to\;wash\;water\;\left(g\right)}{Mass\;of\;analyte\;released\;from\;1\;g\;cabbage\;to\;exudate\;\left(g\right)}\times 100=\frac{c\;\left(analyte\;in\;wash\;water\right)\times \frac{3,000}{3,600}}{c\;\left(analyte\;exudate\right)\times \frac{450}{500}}\times 100=\frac{c\;\left(analyte\;in\;wash\;water\right)}{c\;\left(analyte\;in\;exudate\right)}\times 97$$ (1)

### Water quality analyses

Frozen samples were thawed and diluted. The pH was measured using a pH meter (Oakton Instruments, Vernon Hills, IL, USA). Total dissolved solids (TDS) were measured using a TDS meter (Model 135A; Thermo Orion, Germany) [4]. Chemical oxygen demand (COD) was determined following Hach method 10236 [12]. Absorbance (UV_254_) was measured using a UV-Vis spectrophotometer (Model DU 730, Beckman Coulter, NJ, USA), after filtering the samples through a membrane filter (0.45 μm, VWR International, Radnor, PA, USA) [10].

The CLD of all samples equilibrated at 4°C for 30 min was analyzed by the HACH method [13]. Prior to the measurement, frozen cabbage exudate and wash water were thawed and standardized by diluting with phosphate buffer (pH 6.5, 50 mM) to reach a COD level of 1,500 mg/L. This treatment was performed to ensure a same initial FC/COD ratio, which has a significant impact on the rate of chlorination. Concentrated sodium hypochlorite solution was added to each sample to achieve approximately 1,000 mg/L FC, as measured by the *N*,*N*-diethyl-*p*-phenylenediamine (DPD) method [13]. The reaction mixtures were then vortexed for 30 s and incubated at 4°C for up to 2 hr. Samples were drawn at preset intervals (0, 2, 5, 15, 30, 60, 90, 120 min) and tested for the residual FC by the DPD method. The CLD was calculated as CLD = FC_0_—FC_t_, where FC_0_ denotes the FC level at time zero and FC_t_ indicates that at a specific time interval.

### Quantification of sugars, organic acids, and phenolics

Sugars, organic acids, and phenolic acids were characterized using an Agilent Infinity 1260 HPLC system (Agilent Technologies, Santa Clara, CA, USA), equipped with a 1260 Infinity II Refractive Index Detector and 1260 Infinity II Diode Array Detector. All compounds were identified and quantified with calibration curves established using external standards.

The sugar profile was determined using a slight modification of a solid-phase extraction method followed by HPLC as described by [14]. Freeze-dried samples were dispersed in distilled water, filtered with a 0.45 μm syringe filter (VWR International, Radnor, PA, USA), and then applied to a Sep-Pak C-18 cartridge (Waters Ltd., Mississauga, Ontario, Canada). The eluate was concentrated under nitrogen and filtered through a 0.2 μm membrane filter (VWR International). Samples (20 μL) were injected onto a Phenomenex RPM monosaccharide column (Phenomenex, Torrance, CA, USA) and eluted isocratically with HPLC-grade water at 1 mL/min; the column temperature was 85°C.

Organic acids were quantified following a modified method from [15]. Freeze-dried cabbage exudate or wash water samples (90 mg) were dispersed in 1.5 mL of solvent consisting of 20 mM monobasic potassium phosphate (KH_2_PO_4_) and 247 mM methanol in water. The resulting suspension was acidified to pH 2.0 using phosphoric acid, vortexed, centrifuged, and passed through a 0.2 μm syringe filter (VWR International). The filtrate (20 μL) was injected onto a Phenomenex C18 column (4.6 x 250 mm, 5 μm) and eluted isocratically with the solvent described above, except that pH was adjusted to pH 2.4 using phosphoric acid. Elution was performed at a flow rate of 0.3 mL/min and column temperature of 30°C. The organic acids were detected with a UV detector at wavelength of 210 (for citric and malic acid) and 245 nm (for oxalic acid).

Total phenolic content (TPC) was measured using the Fast Blue BB (FBBB) assay [16] which is less affected by non-phenol compounds, such as reducing sugars and ascorbic acid, as compared to the Folin–Ciocalteu method [17]. Phenolic acids were extracted and analyzed using the method by [18] with slight modification. In brief, freeze-dried samples were dispersed in methanol/acetone/water (7/7/6, v/v/v) and centrifuged (9,000 x *g*, 5 min). Free phenolic acids were extracted with ether/ethyl acetate (1/1, v/v) after acidifying the supernatant. Conjugated phenolic acids were enriched with ether/ethyl acetate after hydrolyzing and acidifying the remainder from the first extraction. Finally, the precipitate resulting from centrifugation was hydrolyzed, re-centrifuged, acidified, and extracted for insoluble phenolic acids. All ether/ethyl acetate extracts were dried under nitrogen, re-dissolved in 1 mL methanol and filtered using a 0.2 μm syringe filter. Filtrates were analyzed on a Phenomenex C18 column (250 mm × 4.6 mm) at a flow rate of 0.1 mL/min. A linear, 42-min gradient mobile phase was used: 90% solvent A (acetic acid/H_2_O, 2:98, v/v) and 10% solvent B (acetic acid/acetonitrile/H_2_O, 2:30:68, v/v/v) to 100% solvent B.

### Characterization of proteins and peptides from cabbage wash water

Crude protein was measured by a Total Kjeldahl Nitrogen (TKN) assay using Hach method 10242 [19] and was partially extracted from the cabbage wash water as follows. Freeze-dried samples obtained from 45 mL of wash water were reconstituted with water to 22.5 mL, and 15 mL of the resulting dispersion was drawn and centrifuged at 15,000 x *g* for 10 min to remove insoluble matter. The supernatant was centrifuged (5,000 x *g* for 30 min) in a Macrosep centrifugal dialysis tube (Pall Corp., Port Washington, New York, USA) with molecular weight cutoff (MWCO) of 30 kDa. The filtrate was subjected to a second centrifugation under the same condition in tubes with MWCO of 3 kDa. The retentates obtained from these two centrifugation steps were combined and restored to 15 mL with water. The resulting dispersion was defined as the high-MW fraction, which contained mostly proteins/peptides according to the TKN test (S1 Table).

The filtrate obtained after the second centrifugation, defined as the low-MW portion, was further acidified with formic acid (0.2% v/v) and fractionated by solid phase extraction, using Waters Sep-Pak C-18 cartridges (Waters Ltd., Mississauga, Ont., Canada). Prior to use, the cartridges were conditioned with pure methanol and equilibrated with 0.2% formic acid (v/v) in water. Filtrate (2 mL) was loaded onto a conditioned cartridge and eluted with 4 mL water containing 0.2% formic acid, followed by a series of methanol/water mixtures (all containing 0.2% formic acid) with methanol content increasing from 5% to 90%. Eluates were pooled based on their absorbance at 280 nm (OD_280_) and chemical composition.

### COD and CLD of identified and model compounds

Organic compounds including malic, citric, and oxalic acid, glucose, and fructose, were dissolved in phosphate buffer at their actual contents in the standardized wash water with COD of 1,5000 mg/L. Soy protein acid hydrolysate (modelling low-MW, high-polarity peptides), and gallic acid (modelling phenolics) were dissolved in phosphate buffer at the measured contents of compounds they modelled. The reason for choosing those two model compounds will be elaborated in the section of ‘Chemical composition of cabbage exudate and wash water’. Two fractions of protein (high-MW, and low-MW, low polarity) obtained in the section of ‘Characterization of proteins and peptides from cabbage wash water’ were tested for COD as is without further dilution. All samples were tested for COD as described in the section of ‘Water quality analyses’.

For CLD measurement, the abovementioned compounds were dispersed in phosphate buffer at various concentrations including their actual or modelled contents in the standardized wash water. The resulting dispersions were tested for CLD at different time intervals as described in the section of ‘Water quality analyses’.

### Statistics

All treatments and assays were performed in triplicate. Data in tables are presented as mean ± standard error. Regression models (linear, polynomial, logarithmic, power, exponential, and Weibull) were tested for predicting CLD by substrate concentration.). The best model with the lowest root mean square error (RMSE) and Akaike information criterion (AIC) was chosen.

## Results and discussion

### Water quality parameters of cabbage exudate and wash water

The average COD, UV_254_, TDS, and CLD_120_ (CLD at 120 min) of cabbage exudate and of diced cabbage wash water are shown in Table 1. The wash water was prepared in a manner similar to industrial fresh produce processing and afforded a similar organic load (typically over 8,000 mg/L COD for diced cabbage) as has been reported [4]. The UV_254_, used to estimate CLD [10], was relatively high in the 20-fold dilution, indicating a high content of proteins, peptides, and/or phenolic compounds; the CLD of 2,000 mg/L was two to three times higher than reported for lettuce or spinach [20].

**Table 1:** Water quality parameters of cabbage exudate and wash water.

Parameter ^†^	Exudate	Wash water	Conversion factor
pH	6.6 ± 0.2	6.5 ± 0.2	NA^**‡**^
COD (mg/L)	64,300 ± 1,000	6,060 ± 70	9.1%
TDS (mg/L)	79,600 ± 4750	890 ± 24	10.8%
UV254_adj_	0.93 ± 0.06	0.10 ± 0.01	10.5%
CLD_120min_ (mg/L)	21,300 ± 1,300	2,000 ± 100	9.1%

^†^ The UV_254_ was measured after diluting the samples 20 times. Values for all other parameters represented for the samples without dilution.

^**‡**^ NA: not applicable.

### Chemical composition of cabbage exudate and wash water

The chemical composition of the cabbage exudates and wash waters is shown in Table 2. Cabbage tissue exudate contain primarily sugars, followed by proteins/peptides, organic acids, and phenolic acids. Although cabbage tissues also contain dietary fibers, they are insoluble in water and have limited contribution to CLD and are not included in these analyses.

**Table 2:** Chemical composition of cabbage exudate and wash water.

Chemicals (in mg/L)	Exudate	Wash water	Conversion factor
***Total solid content***	79,200 ± 1200	7,210 ± 620	8.8%
***Sugars***			
Glucose	25,600 ± 1,280	2,730 ± 230	10.3%
Fructose	26,500 ± 1,100	2,310 ± 109	8.5%
***Proteins/peptides***			
Total protein/peptides	14,100 ± 3,350	650 ± 47	4.5%
Insoluble	NA^†^	129 ± 13	NA
Soluble	NA	512 ± 22	NA
MW<3 kDa	NA	355 ± 11	NA
High polarity	NA	67 ± 3	NA
Low polarity	NA	288 ± 9	NA
MW>3 kDa	NA	156 ± 3	NA
***Organic acids***			
Citric	1,730 ± 30	154 ± 15	8.7%
Malic	1,230 ± 70	129 ± 16	10.2%
Oxalic	414 ± 13	53 ± 7.3	12.4%
***Phenols*, *total***			
Total phenolic content	769 ± 22	55.0 ± 2.4	6.9%
Soluble	162 ± 15	16.2 ± 1.5	9.7%
Insoluble	606 ± 30	38.5 ± 2.7	6.2%
***Phenolic acids*, *free***			
Caffeic acid	0	0	NA
Chlorogenic acid	0	0	NA
Ferulic acid	0.60 ± 0.05	0.01 ± 0.00	1.6%
p-coumaric acid	0.01 ± 0.00	0.01 ± 0.00	97%
Sinapic acid	6.34 ± 0.29	0.08 ± 0.01	1.2%
***Phenolic acids*, *conjugated***			
Caffeic acid	1.87 ± 0.37	0.16 ± 0.05	8.3%
Chlorogenic acid	0.51 ± 0.04	0.05 ± 0.01	9.5%
Ferulic acid	3.89 ± 0.12	0.28 ± 0.08	7.0%
p-coumaric acid	0	0	NA
Sinapic acid	19.8 ± 2.6	1.49 ± 0.11	%
***Phenolic acids*, *bound***			
Caffeic acid	0	0.07 ± 0.02	NA
Chlorogenic acid	0	0	NA
Ferulic acid	0.07 ± 0.03	0.03 ± 0.01	41.6%
p-coumaric acid	0	0.02 ± 0.00	NA
Sinapic acid	2.19 ± 0.19	0.33 ± 0.00	14.6%

^†^ NA: not applicable.

Among all compounds identified in cabbage exudate and wash water, sugars contributed to over 70% of the total solids content, primarily as glucose and fructose in similar molarities. Sucrose was not detected in either the exudate or wash water, although other studies have shown its presence in cabbage at low concentration [21].

Proteins and peptides were the second most abundant class of chemicals in the cabbage exudate and wash water, making up 17.8% and 9.0% of the total solid contents respectively. Water-soluble proteins accounted for 80% of the proteins in wash water, with high MW (above 3 kDa) and low MW (below 3 kDa) contributing to approximately 30% and 70% of the total soluble proteins. The low MW proteins were further separated into two fractions: a high-polarity fraction representing 10.3% of the total proteins, together with sugars and organic acids, and a low-polarity fraction comprising 44.3% of the total proteins. The high-polarity fraction was modelled by soy protein hydrolysate in the following studies, due to the interference from coexisting sugars and organic acids. Soy protein hydrolysate are low-MW mixtures and share similar contents of lysine and cysteine, two most reactive amino acid residues, with the low-MW cabbage proteins [22].

Organic acids make up approximately 4.7% of the total solid content of the cabbage wash water, primarily in the forms of citric acid (2.1% of total solid content), malic acid (1.8%), and oxalic acid (0.7%). These acids, together with other negatively charged compounds, contributed collectively to a mildly acidic pH (6.2~6.5) of the cabbage wash water. Organic acids migrated into water at higher rates during washing than sugars and proteins/peptides, evidenced by their greater conversion factors.

The total phenolic content accounted for approximately 1.0% and 0.7% of the total solid content in cabbage exudate and wash water, respectively. Only 29% of those phenolic compounds were water-soluble, representing 0.2% of the solid content in the cabbage wash water. Phenolic acids, the major reactive form of phenolics [11], were further profiled by HPLC. Over 95% and 85% of the phenolic acids in cabbage exudate and wash water, respectively, were detected as water-soluble and conjugated molecules. Sinapic and ferulic acids were the major species of phenolic acids. However, the phenolic acids identified in this study represented less than 2.5% of the total phenolic content, which was comparable to a previous study on cabbage [23]. As such, the COD and CLD of phenolics were modelled by gallic acid at a concentration equal to the total phenolic content.

### Chemical oxygen demand in wash water and its constituents

**Fig 1:**
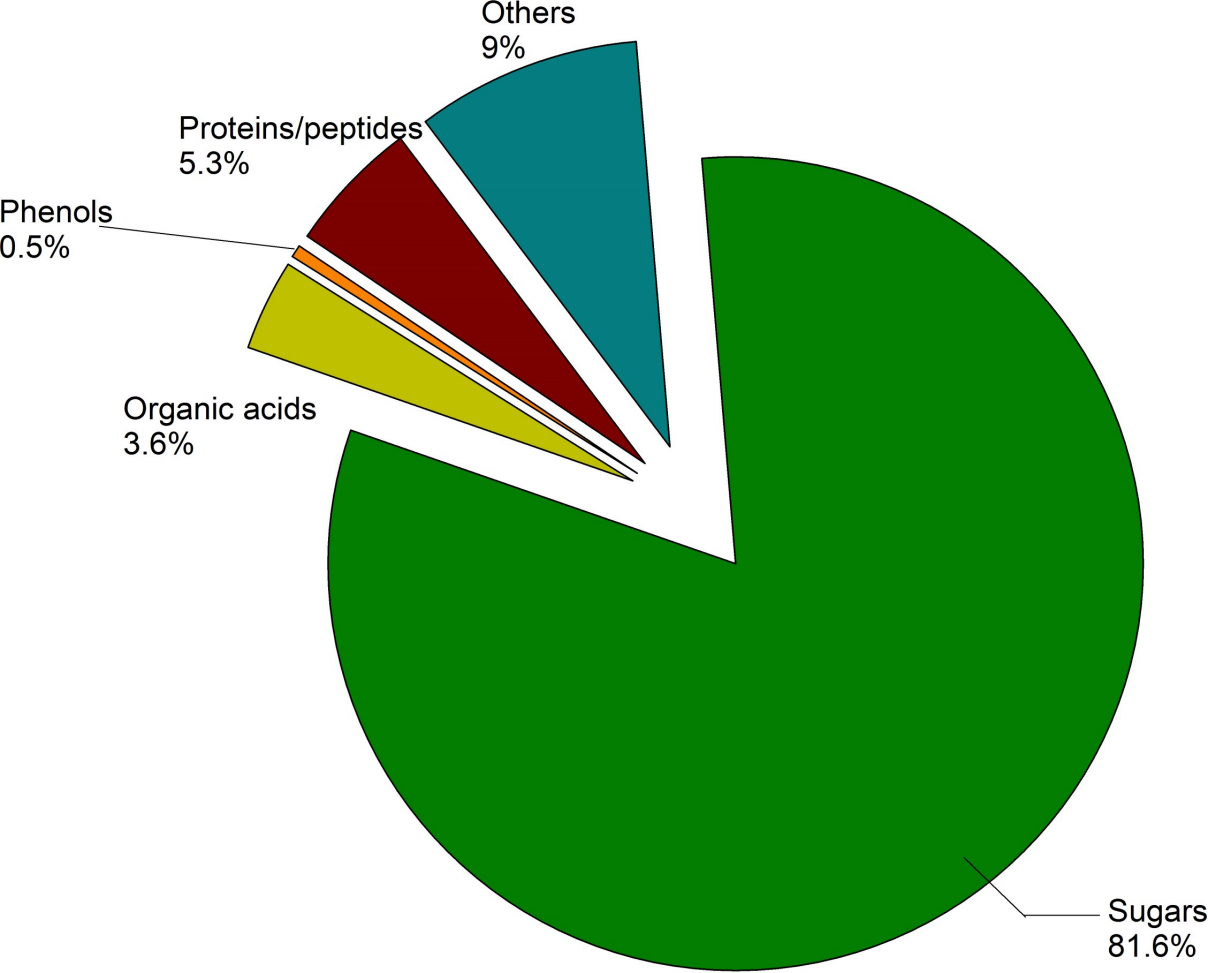
Contribution to chemical oxygen demand (COD) by constituents of cabbage wash water.

The COD of cabbage exudate, wash water, and their constituents are presented in Fig 1. All measurements were performed without prior chlorination, as the effect of chlorination on the COD of wash water samples was negligible (S2 Table). Sugars, proteins and peptides, organic acids, and phenols contributed to 91% of the total COD in cabbage wash water. Sugars including glucose and fructose accounted for 82% of the COD. Proteins and peptides contributed to 5.3% of the COD in cabbage wash water. Organic acids (3.6%) and phenols (0.5%, represented by gallic acid) contributed slightly to the total COD as well.

### Time and concentration dependent CLD of cabbage wash water and its constituents

**Fig 2:**
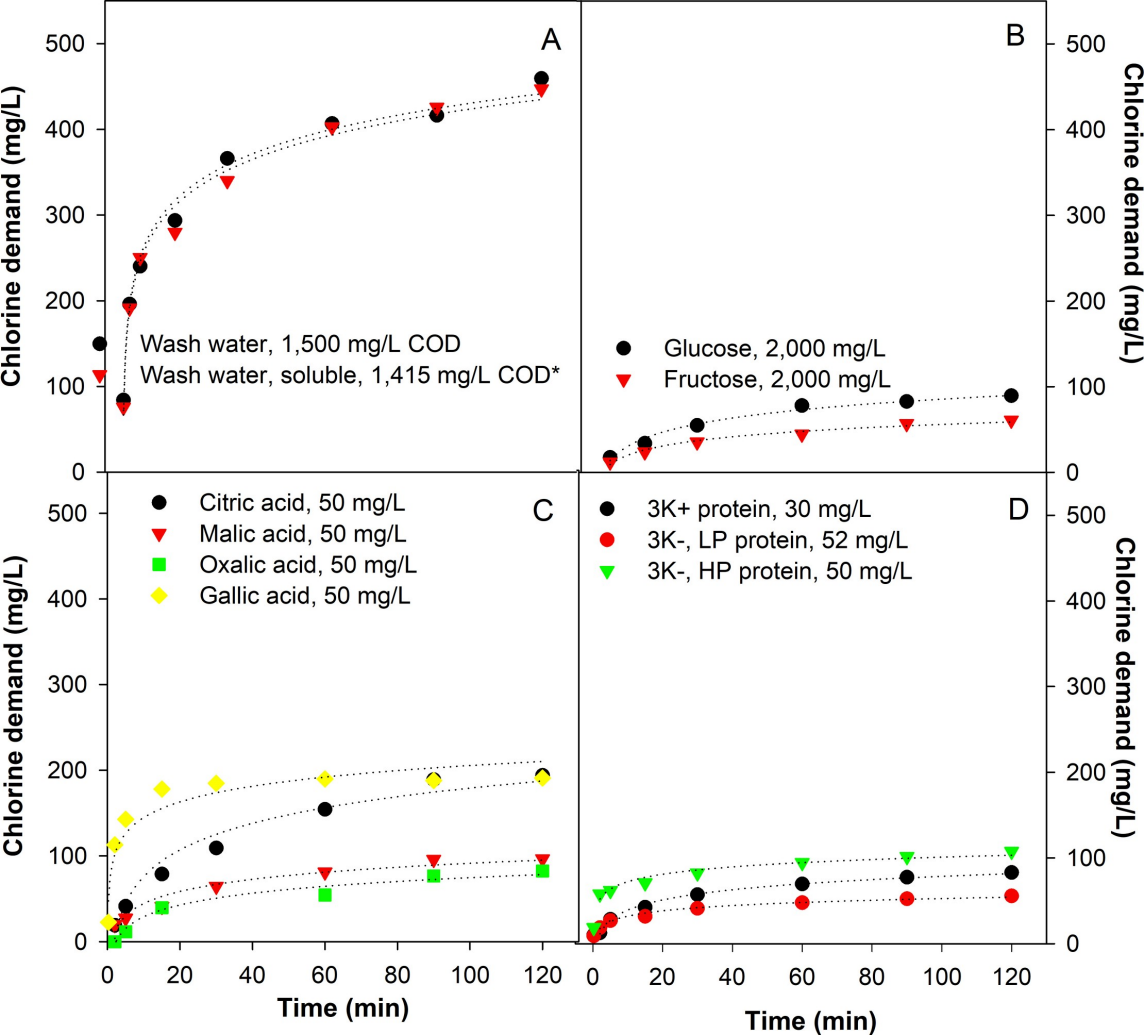
Time- and concentration-dependent chlorine demand (CLD) of different compounds identified in this study. Fig 2A–2D show the time-dependent CLD of cabbage wash water (A) and its constituents: sugars (B), organic acids and phenols (C), and proteins (D). The concentration of compounds shown in the figures represent the approximate concentrations in the standardized wash water (COD = 1,500 mg/L). Data for more concentrations are available in S1 Fig. Fig 2E and 2F show the concentration-dependent chlorine demand (CLD) of different compounds identified in this study. Chlorination time was 15 (left) and 120 (right) min. Values on x-axis denote for substrate concentration. Data for more times are available in S2 Fig.

The time-dependent CLD of organic compounds or their model compounds under conditions and time frames relevant to the industrial cabbage processing are presented in Fig 2. The CLD of cabbage wash water accumulated over time, but rapidly increased within the first 5 min, followed by a more gradual increase towards the end of 120 min (Fig 2A). The CLD of cabbage wash water in the first five minutes accounted for 49% of that achieved within the entire 120 min. In addition, soluble compounds separated by centrifugation contributed to over 95% of the CLD in the wash water.

Fig 2B–2D show the time-dependent CLD of various compounds at same concentration (50 mg/L), except for sugars (2,000 mg/L). Additional data on other concentrations of substrates are available in S1 Fig. Among the compounds tested, the CLD increased in the following order: glucose<fructose<<oxalic acid <malic acid<soy protein hydrolysate<citric acid<gallic acid. Notably, the CLD of 50 mg/L gallic acid was 2.5 times higher than that of 2,000 mg/L fructose. More importantly, the pattern of CLD increase varied greatly among different compounds. For instance, sugars (Fig 2B) showed only one seventh of the maximum CLD in the same period, and the reaction proceeded gradually. Conversely, gallic acid (Fig 2C) reacted with FC rapidly in the first 5 min, achieving 80% of the CLD measured at 120 min. Finally, the CLD of proteins depended on their MW and polarity, with low MW and high polarity fractions (‘3k-, HP’, modelled by soy protein hydrolysate) exhibiting the highest CLD.

Fig 2E and 2F shows the concentration-dependent CLD of different compounds at 15 min and 120 min. Additional figures obtained at more time points are shown in S2 Fig. Under all tested conditions, a quadratic relationship was found between concentration and CLD for the compounds investigated in this study. Gallic acid exhibited the highest CLD per mg/L concentration, followed by proteins/peptides and organic acids, and sugars that were one to two orders of magnitude less reactive. However, a more pronounced increase in CLD from 15 to 120 min was observed with organic acids and sugars, which suggested a more continuous reaction pattern and significant long-term CLD of those compounds. The terms used for predicting the CLD attributable to each type of sugar, organic acid or model compound are shown in Table 3, with the corresponding *R*^*2*^ for each compound at 15 and 120 min. Results at more time intervals are available as S3 Table.

**Table 3:** Quadratic models derived for predicting chlorine demand (y, in mg/L) of different chemical compounds by their concentrations (x, in mg/L). The models are written as y = ax^2^ + bx + c.

Compound	Range of x	y in 15 min				y in 120 min			
		a	b	c	R^2^	a	b	c	R^2^
Glucose	1000~8000	-4 x 10^−7^	0.008	3.662	0.960	-2 x 10^−6^	0.053	2.216	0.971
Fructose	1000~8000	-1 x 10^−7^	0.006	8.614	0.890	-2 x 10^−6^	0.058	2.438	0.971
Citric acid	50~400	9 x 10^−4^	0.194	71.35	0.904	-2 x 10^−4^	1.881	66.00	0.912
Malic acid	100~500	1 x 10^−4^	0.931	-18.13	0.972	1 x 10^−4^	0.686	22.17	0.962
Oxalic acid	100~500	-3 x 10^−5^	0.301	-1.333	0.909	-6 x 10^−4^	0.740	39.20	0.971
SPH*	10~200	-3 x 10^−3^	2.435	1.542	0.988	2 x 10^−3^	1.504	67.04	0.991
Gallic acid	10~200	-1 x 10^−4^	2.748	18.16	0.986	-2 x 10^−3^	2.498	60.20	0.993

* SPH: soy protein hydrolysate.

### Contribution to CLD by identified compounds in cabbage wash water

**Fig 3:**
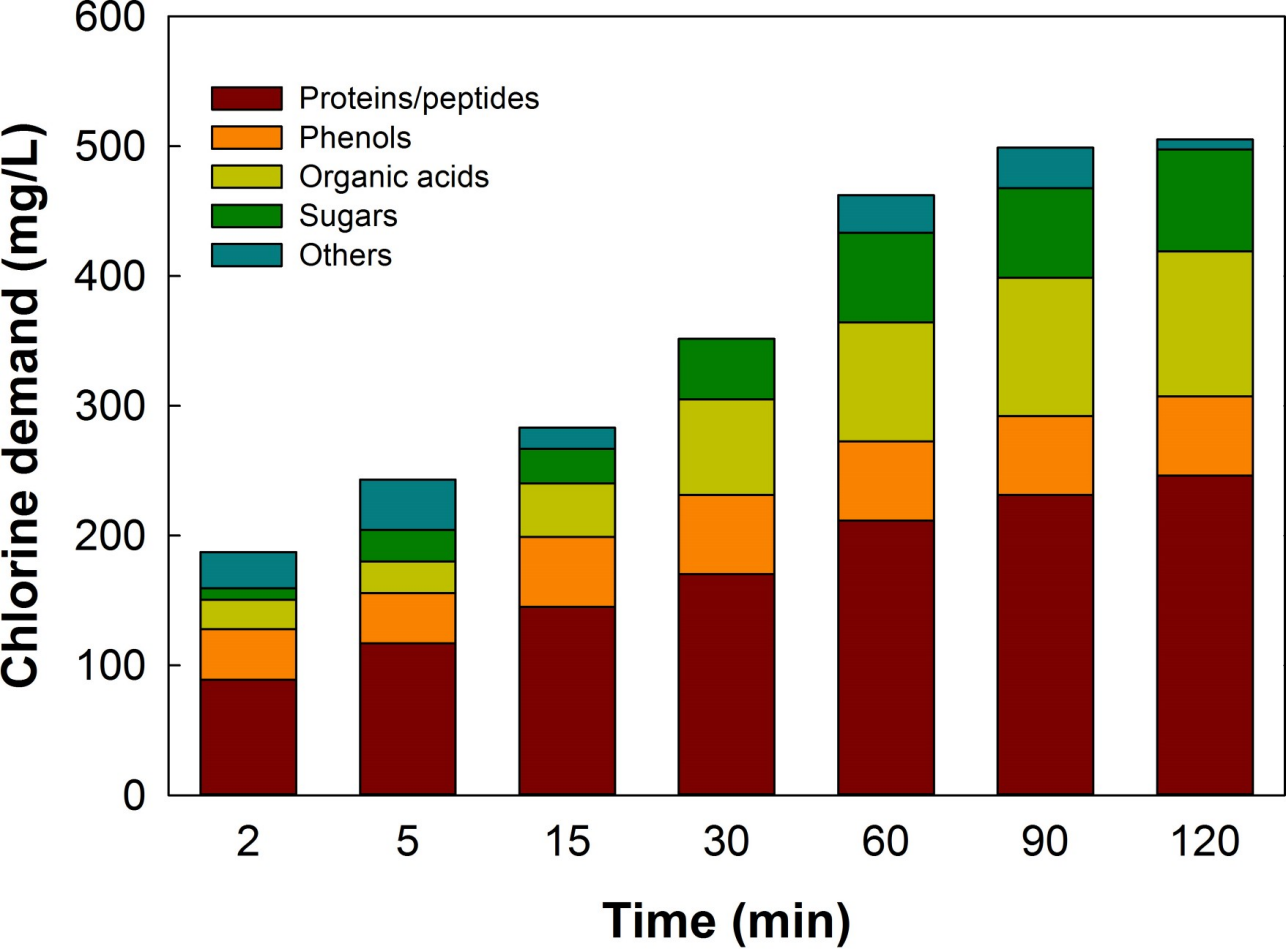
Time-dependent contribution to chlorine demand (CLD) by different compounds in cabbage wash water.

By combining the results in the above two sections, we predicted the CLD of various compounds in cabbage wash water at a given time. Specifically, the concentration of each identified or model compound discussed in the section of ‘Chemical composition of cabbage exudate and wash water’ was applied to its quadratic function illustrated in Table 3 and S3 Table. As shown in Fig 3, all the compounds investigated in this study accounted for 85% of the total CLD in the first 5 min of chlorination and over 94% of that from 15 to 120 min. Among those compounds, proteins and peptides accounted for about a half of the total CLD throughout the washing process. In comparison, organic acids consumed FC more gradually, contributing to 12% and 22% of the total CLD at 2 and 120 min, respectively. Phenols represented by gallic acid accounted for over 20% of CLD in the first two minutes, but their CLD increased less significantly compared to those of other compounds, probably due to their rapid depletion. Therefore, the percentage of contribution by phenols diminished continuously as chlorination proceeded. Lastly, sugars ranked the lowest in CLD despite their highest content. The CLD of sugars was quite low at the beginning of chlorination, but it increased continuously and was 15% of the total CLD after 2 hr.

## Discussion

Chemical composition determines the COD and CLD of the fresh produce wash water, the pivotal indices for its quality. The abundance of a compound in the water depends on two factors: its prevalence in the produce, and its diffusion rate determined by its physical and chemical properties. Chemicals present in exudate reflect their abundance in cabbage plant tissues, while their contents in wash water are influenced by both of their abundance in cabbage and their solubility in water. Sugars made up over 70% of the total solids content in the cabbage wash water due to their abundance in tissues and high solubility as of result of low MW and high polarity. Proteins and peptides abound in the exudate but exhibited relatively low CF (4.6%), possibly due to the bulky structure and limited water solubility [24]. Our further investigation revealed the predominance of soluble, low-MW fractions of proteins and peptides. In addition, most of the soluble proteins were expected to carry net negative charges, given that 86% of the soluble proteins precipitated at pH 4.6 (S4 Table). Organic acids are smaller, charged molecules and exhibited relatively high CF, but they ranked only third in abundance in the wash water, due to its much lower content in the exudate compared to proteins. Finally, phenolics were found at the lowest level among all investigated compounds, which was attributable to their low solubility and low content in vegetables. The few types of phenolic compounds identifiable by HPLC suggests underrepresentation of total phenolics in wash water by the HPLC results. Therefore, the COD and CLD of phenols were modelled by gallic acid at its equivalent concentration measured by FBBB in this study.

The COD of a compound links directly to its content and chemical formula. It could be predicted by considering complete oxidation of all carbon, hydrogen, nitrogen, and sulfur atoms, and the actual COD of different compounds measured in our study matched the prediction. The ranking of COD contribution was the same as that of the total solids content contribution, with sugars being the highest and phenols being the lowest.

The CLD of a compound in wash water is determined by its concentration and reactivity with chlorine. For chlorination which belongs to electrophilic reaction, the reactivity of a substrate is attributable to two factors: maximal capacity for chlorination (thermodynamic) dependent on the number of unpaired electrons, and rate constant (kinetic) associated with the electronegativity of functional groups [25].

In this study, the changes in CLD of cabbage wash water was biphasic: a rapid growth in the first 15 min, followed by a slow, sustained accumulation for the next 105 min. This was attributed to the different reaction rates of the various compounds released from the cabbage and was reflected in the FC consumption in the two phases. Initially, phenols represented by gallic acid reacted with FC rapidly in the first 5 min, contributing to over 20% of total CLD. This was due to the high reactivity of aromatic rings substituted with hydroxyl groups [25]. However, the phenols were low in abundance and were consumed rapidly, and thus diminishing their contribution to CLD relative to other compounds. It should be noted that other phenolic species with different substitution groups than gallic acid may exhibit different rate constants and will have different CLD patterns. Organic acids and sugars exist in greater abundance compared to the phenols and should have a higher CLD, but they also react more slowly due to their functional groups (carboxyl for acids and ketone/aldehyde for sugars) which would lead to a much lower CLD at early stages of chlorination compared to phenols [8], followed by a steady increase in CLD afterwards. Finally, proteins and peptides were also major reactants with FC, accounting for over 50% of the CLD throughout the 120-min period. Proteins and peptides vary substantially in structural composition, possessing diverse functional groups including amines, thiols, aromatic rings, and carboxyl groups, which together with the variation in MW and polarity of the proteins, necessarily result in unique rate constants. This was observed in our study where the proteins and peptides reacted with FC at different stages of chlorination.

This study focused on cabbage, but the chemical composition of the different types of produce varies substantially and should result in different CODs and CLDs. For instance, acidic produce such as tomatoes would be expected to have greater CLD. In addition, ascorbic acid exhibits higher reactivity than citric or malic acids [8], so produce with higher ascorbic acid content (e.g., broccoli) [26] should exhibit greater CLD, assuming comparable cutting size. Alternatively, the COD and CLD of lower root commodities, such as carrots, would be expected to be lower compared to leafy greens because these vegetables contain are less proteins and peptides [22]. Finally, naturally occurring antioxidants such as tocopherols, carotenoids, anthocyanins, and glucosinolates are found in produce at various levels, but are in low abundance. Therefore, the COD contribution of these compounds is expected to be low. In addition, the CLD would also be expected to be low since their concentration is typically as most of them have a limited aqueous solubility and thus a low aqueous diffusion rate. Additional studies are ongoing to identify the source of COD and CLD in other produce varieties, including carrots, onions, Romaine lettuce, and Iceberg lettuce.

The results from this study provide more insight into the unique composition of fresh produce wash water. First, the COD falls in the range of 1,000–10,000 mg O_2_ per liter. This is well above that of typical industrial, municipal, or recreational waste water [27]. Second, the predominance of sugars as a contributor to COD distinguishes wash water from other high-COD waste such as municipal sludge, in which nitrogenous compounds such as proteins contribute mainly to the COD [28]. Third, the wash water provides a full spectrum of nutrients (sugars, proteins or peptides, organic acids, etc.) that is readily available for microorganisms, comparable to industrial or municipal waste stream [29]. Fourth, the CLD of the wash water originates from broad classes of compounds, each of which possesses unique levels and increasing patterns of CLD.

The findings from this study further underscore fundamental factors critical to the development and screening of suitable fresh produce wash water treatment and discharge procedures. Most importantly, COD and CLD arise from distinct types of compounds in the wash water; treatments aimed at effectively treating COD does not guarantee concomitant effectiveness on CLD. In addition, usage of organic acids as acidulants during washing should be reconsidered, and maybe replaced by acids with no CLD such as phosphoric acid. Lastly, cationic absorbent materials may help removal of negatively charged organic acids and proteins/peptides from the wash water, thus lowering its CLD effectively and making it more manageable in reconditioning produce wash water systems.

## Conclusions

Using diced cabbage as a model fresh-cut product, this study identified and characterized major organic compounds and their contributions to chemical oxygen demand and chlorine demand in wash water, an important type of agricultural spent water known for high organic load. Cabbage wash water contains primarily sugars, followed by proteins/peptides, organic acids, and phenols. Sugars, in the forms of glucose and fructose, contribute to the majority (82% of total) of chemical oxygen demand, but play only limited roles in chlorine demand especially in the first 30 min of chlorination. On the other hand, proteins/peptides contribute to 5.3% of chemical oxygen demand, but over 50% of chlorine demand. It is worth noting that not only do protein/peptides have strong reaction rate with chlorine, their contribution to chlorine demand starts immediately upon contact with chlorine and lasts until the end of 120 min testing time. Organic acids contribute to 3.6% of the chemical oxygen demand; they exhibited low initial chlorine demand, which increased continuously to 22% of the total chlorine demand in 120 min. Phenolics play nearly negligible role (0.5%) to the chemical oxygen demand, but a substantial role in chlorine demand, especially immediately upon contact with chlorine. Given that chemical oxygen demand is a critical factor for wastewater treatment while chlorine demand is essential to determine the chlorine replenishment to meet target FC concentration required to prevent pathogen survival in wash water, removing or reducing sugar concentration will lead to significant improvement in waste water treatment and discharge, while removing or reducing protein/peptides will aid significantly in the maintenance of FC concentrations sufficient to ensure safe wash water for fresh and fresh-cut produce processing.

## Supporting information

S1 Fig Time-dependent chlorine demand of selected compounds. (PDF)

S2 Fig Dose-dependent chlorine demand of selected compounds. (PDF)

S3 Fig Representative chromatographs on cabbage juice and wash water for sugar profile analysis. (PDF)

S4 Fig Representative chromatographs on cabbage wash water for a) absorbance at 210 nm (citric and malic acid), and b) absorbance at 246 nm (oxalic acid).
(PDF)

S1 Table Composition of the high-MW fraction obtained in cabbage wash water. (PDF)

S2 Table Effect of chlorination on COD of cabbage wash water diluted to 1,500 mg/L COD. (PDF)

S3 Table Quadratic models for predicting chlorine demand of different compounds by their concentrations. (PDF)

S4 Table Protein/peptide content in different fractions of cabbage wash water. (PDF)
