# Supplementary material for: Impacts and interactions of organic compounds with chlorine sanitizer in recirculated and reused produce processing water
Source: PLoS One. 2018 Dec 12;13(12):e0208945. doi: 10.1371/journal.pone.0208945 (PMC6291160; doi:10.1371/journal.pone.0208945)
Supplement: S1 Fig — (PDF) [file pone.0208945.s001.pdf]

S1 Fig. Time-dependent chlorine demand of glucose (A), fructose (B), citric acid (C), malic acid (D), oxalic acid (E), soy protein hydrolysate (F), and gallic acid (G)

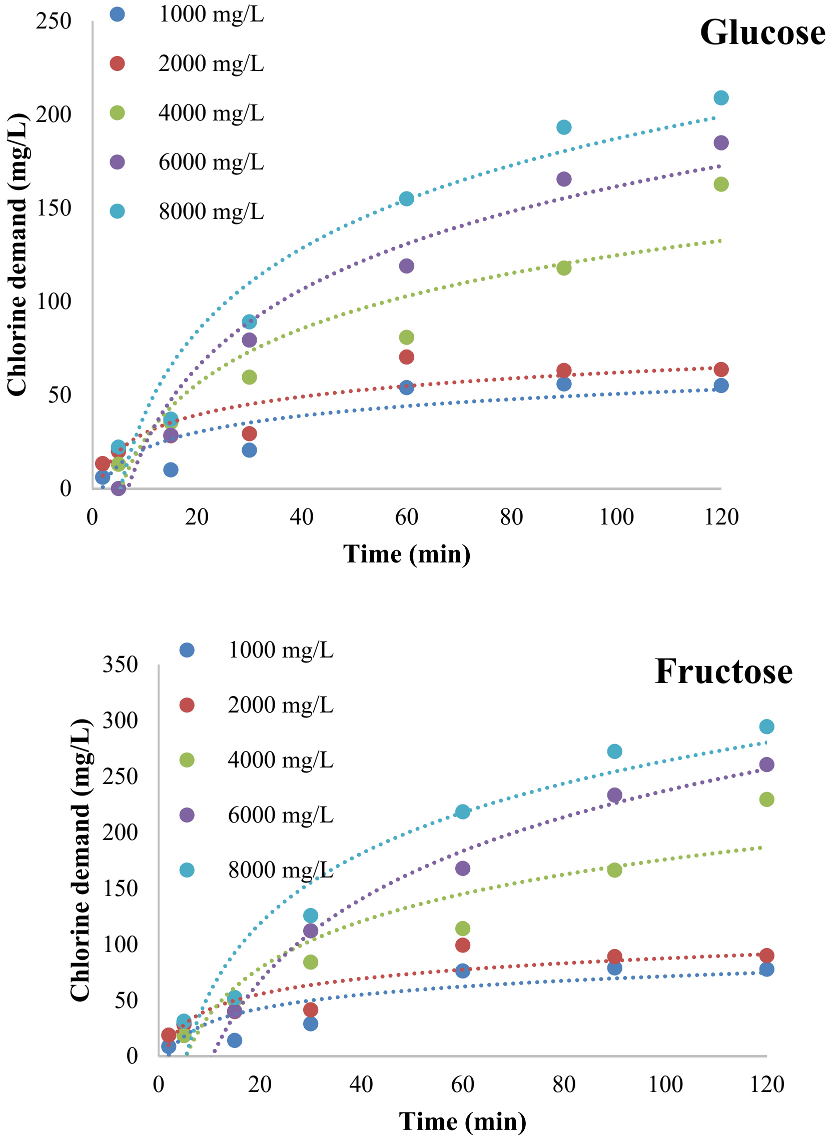

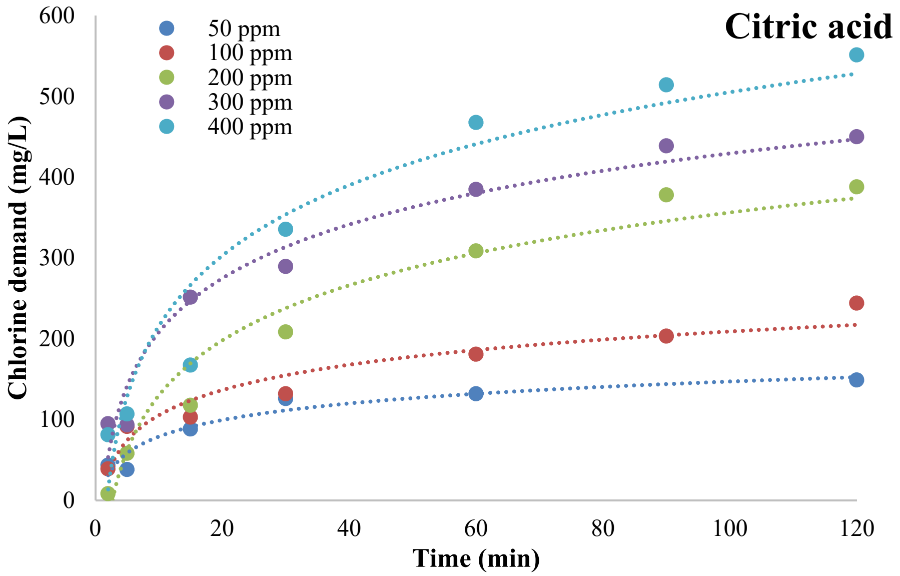
Citric acid
50 ppm
100 ppm
200 ppm
300 ppm
400 ppm
Chlorine demand (mg/L)
Time (min)
0
100
200
300
400
500
600
0
20
40
60
80
100
120

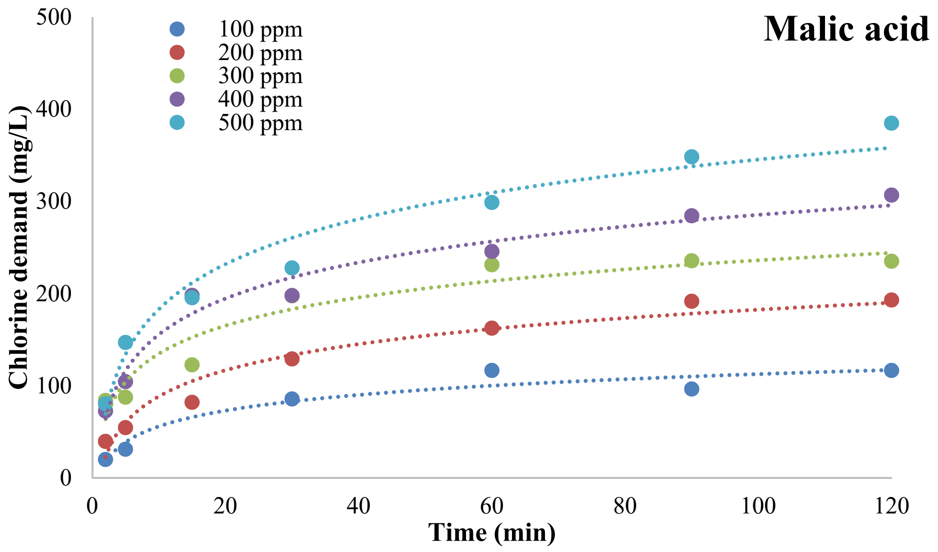
Malic acid
100 ppm
200 ppm
300 ppm
400 ppm
500 ppm
Chlorine demand (mg/L)
Time (min)
0
100
200
300
400
500
0
20
40
60
80
100
120

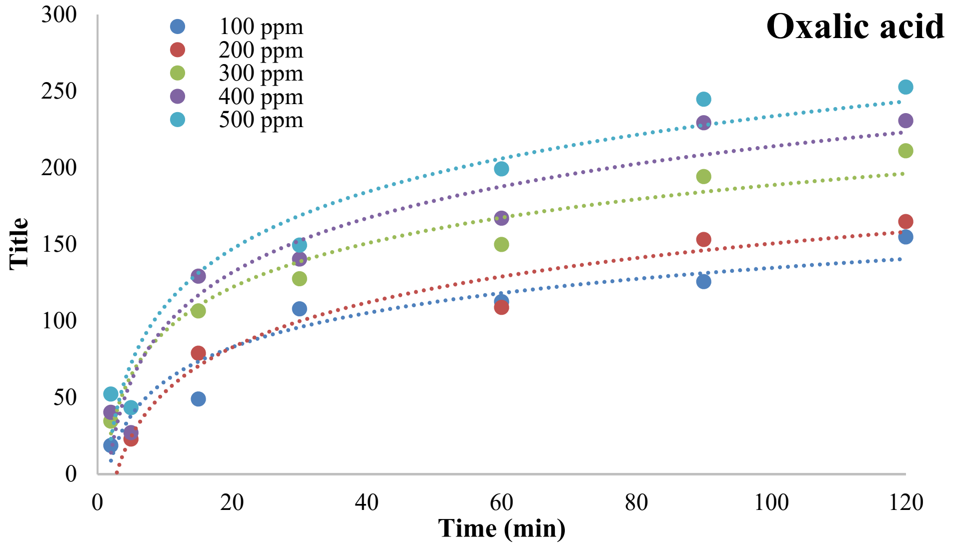
Oxalic acid
100 ppm
200 ppm
300 ppm
400 ppm
500 ppm
Title
Time (min)
300
250
200
150
100
50
0
0
20
40
60
80
100
120

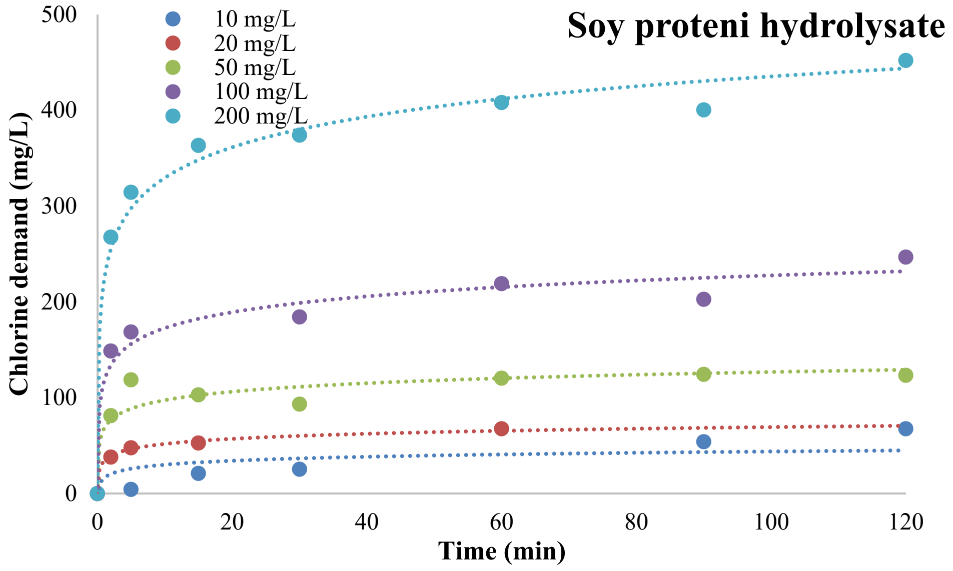
Soy proteni hydrolysate
10 mg/L
20 mg/L
50 mg/L
100 mg/L
200 mg/L
Chlorine demand (mg/L)
Time (min)
500
400
300
200
100
0
0
20
40
60
80
100
120

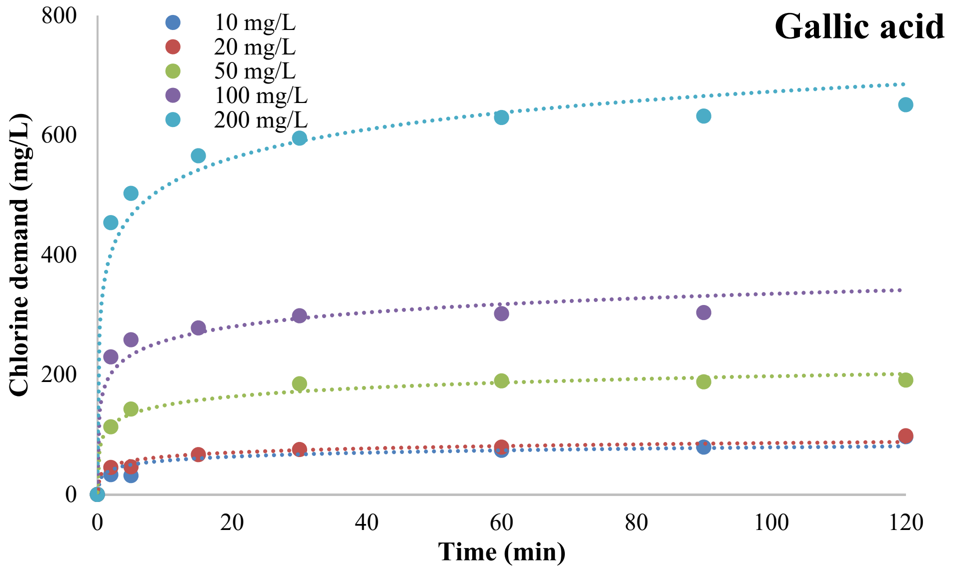
Gallic acid
10 mg/L
20 mg/L
50 mg/L
100 mg/L
200 mg/L
Chlorine demand (mg/L)
800
600
400
200
0
Time (min)
0
20
40
60
80
100
120
