# Supplementary material for: Impacts and interactions of organic compounds with chlorine sanitizer in recirculated and reused produce processing water
Source: PLoS One. 2018 Dec 12;13(12):e0208945. doi: 10.1371/journal.pone.0208945 (PMC6291160; doi:10.1371/journal.pone.0208945)
Supplement: S2 Fig — (PDF) [file pone.0208945.s002.pdf]

S2 Fig. Dose-dependent chlorine demand of glucose (A), fructose (B), citric acid (C), malic acid (D), oxalic acid (E), soy protein hydrolysate (F), and gallic acid (G)

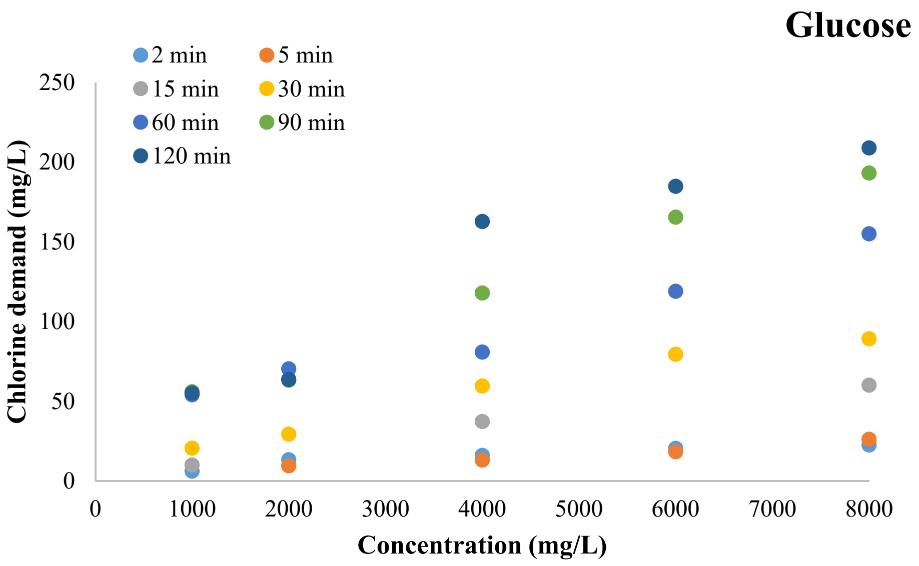

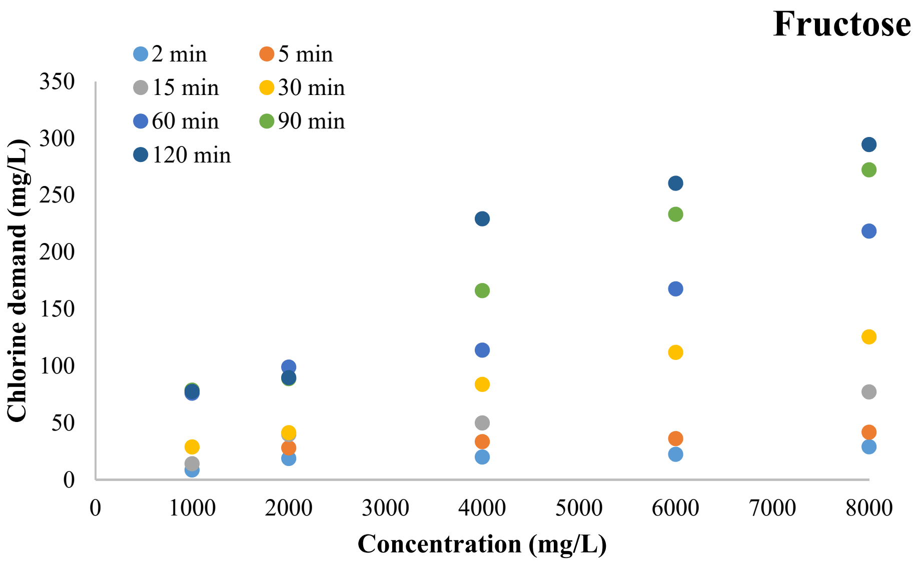

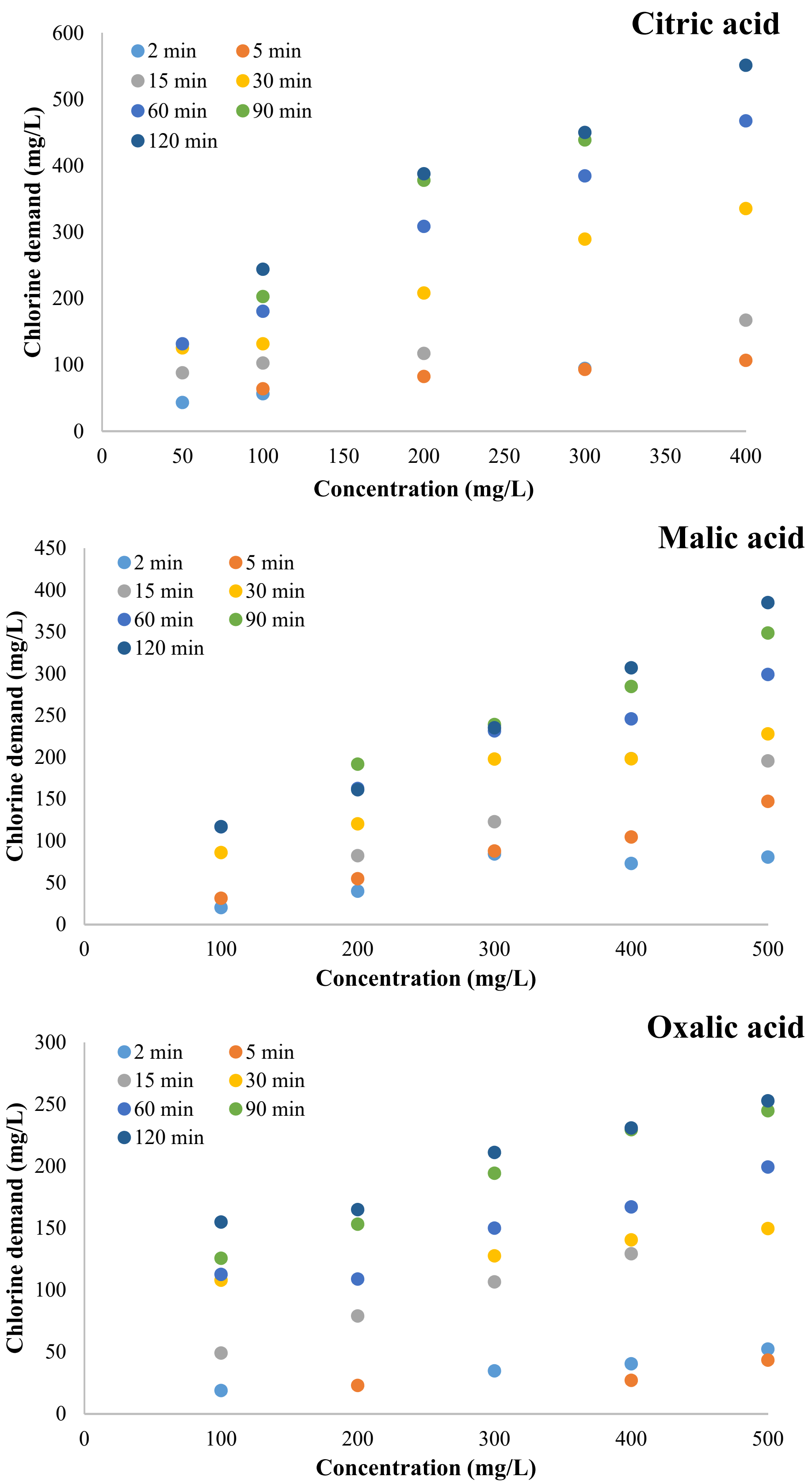
Citric acid
2 min
5 min
15 min
30 min
60 min
90 min
120 min
Chlorine demand (mg/L)
0
100
200
300
400
500
600
0
50
100
150
200
250
300
350
400
Concentration (mg/L)
Malic acid
2 min
5 min
15 min
30 min
60 min
90 min
120 min
Chlorine demand (mg/L)
0
50
100
150
200
250
300
350
400
450
0
100
200
300
400
500
Concentration (mg/L)
Oxalic acid
2 min
5 min
15 min
30 min
60 min
90 min
120 min
Chlorine demand (mg/L)
0
50
100
150
200
250
300
0
100
200
300
400
500
Concentration (mg/L)

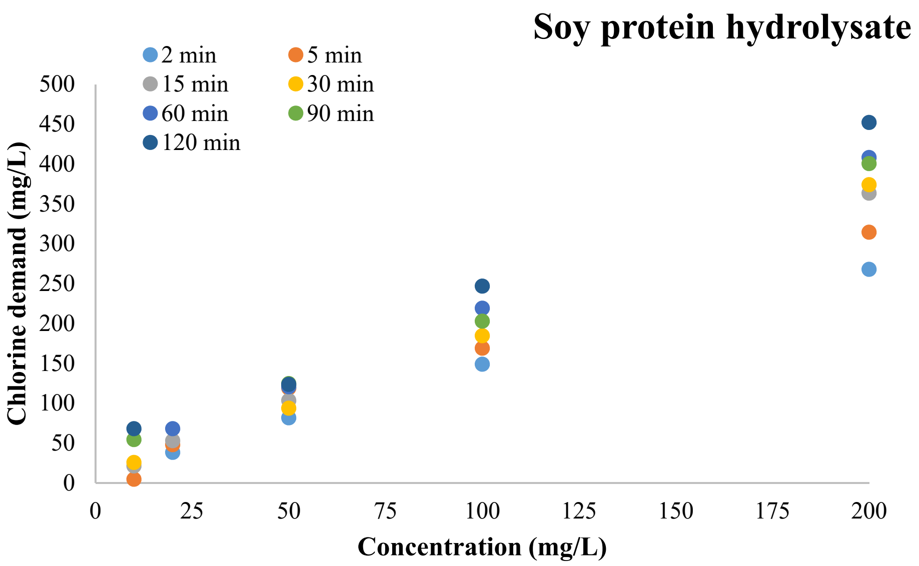
Soy protein hydrolysate
2 min
5 min
15 min
30 min
60 min
90 min
120 min
Chlorine demand (mg/L)
0
50
100
150
200
250
300
350
400
450
500
Concentration (mg/L)
0
25
50
75
100
125
150
175
200

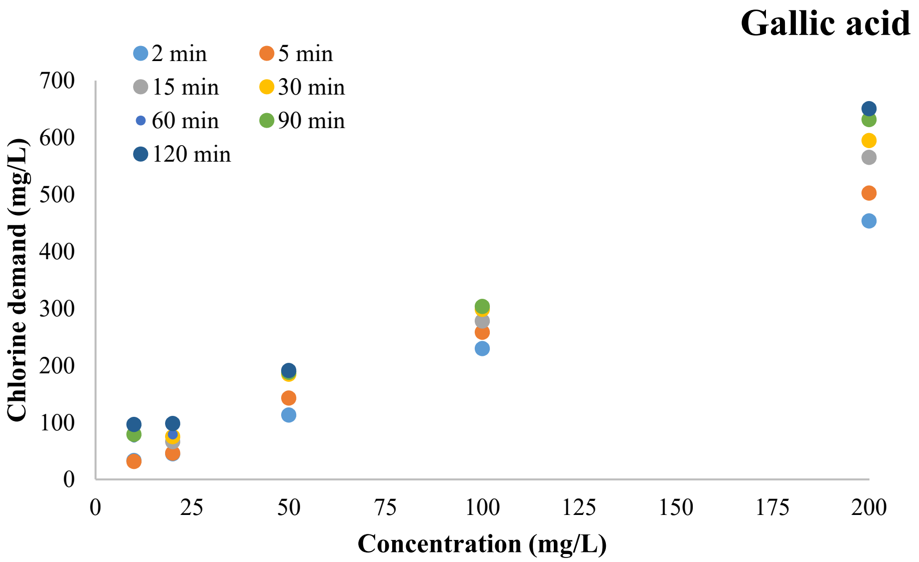
Gallic acid
2 min
5 min
15 min
30 min
60 min
90 min
120 min
Chlorine demand (mg/L)
0
100
200
300
400
500
600
700
Concentration (mg/L)
0
25
50
75
100
125
150
175
200
