# Supplementary material for: Impacts and interactions of organic compounds with chlorine sanitizer in recirculated and reused produce processing water
Source: PLoS One. 2018 Dec 12;13(12):e0208945. doi: 10.1371/journal.pone.0208945 (PMC6291160; doi:10.1371/journal.pone.0208945)

S3 Fig. Representative chromatographs on cabbage juice and wash water for sugar profile analysis

Juice

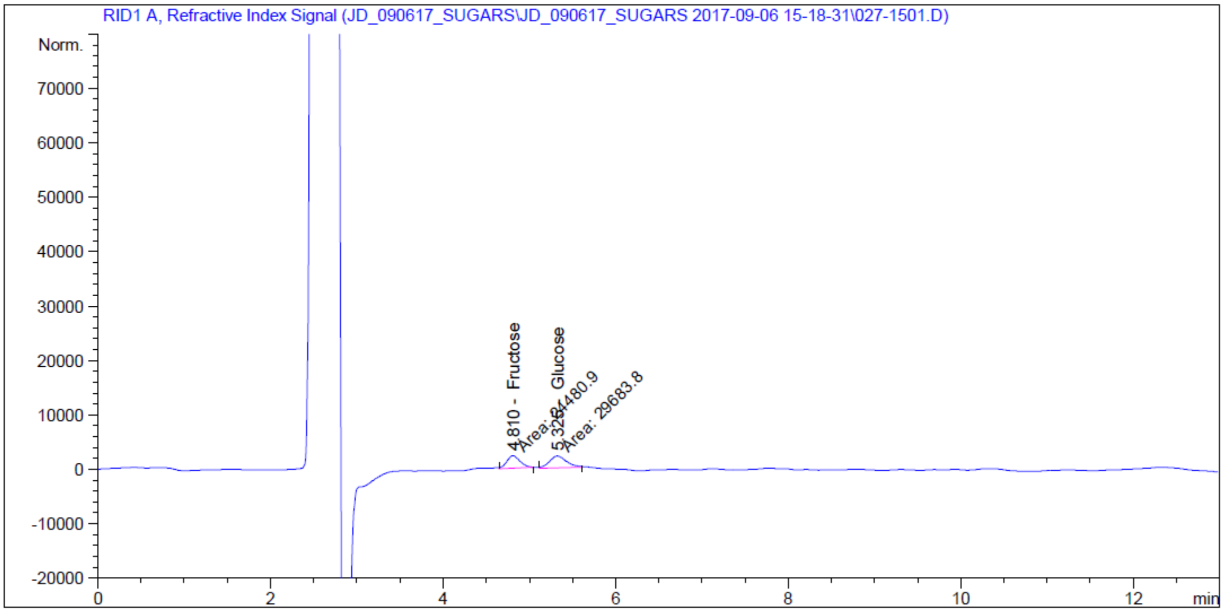

Wash water

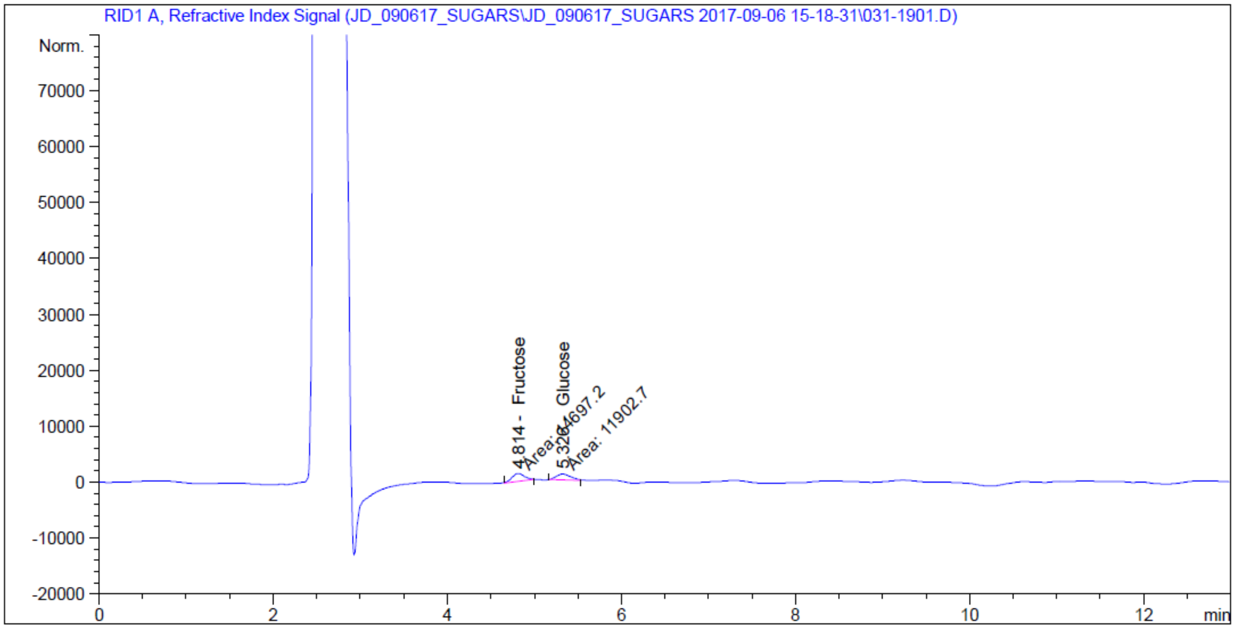

Supplement: S3 Fig — (PDF) [file pone.0208945.s003.pdf]
