# Supplementary material for: Impacts and interactions of organic compounds with chlorine sanitizer in recirculated and reused produce processing water
Source: PLoS One. 2018 Dec 12;13(12):e0208945. doi: 10.1371/journal.pone.0208945 (PMC6291160; doi:10.1371/journal.pone.0208945)

S4 Fig. Representative chromatographs on cabbage wash water for a) absorbance at 210 nm (citric and malic acid), and b) absorbance at 246 nm (oxalic acid)

a)

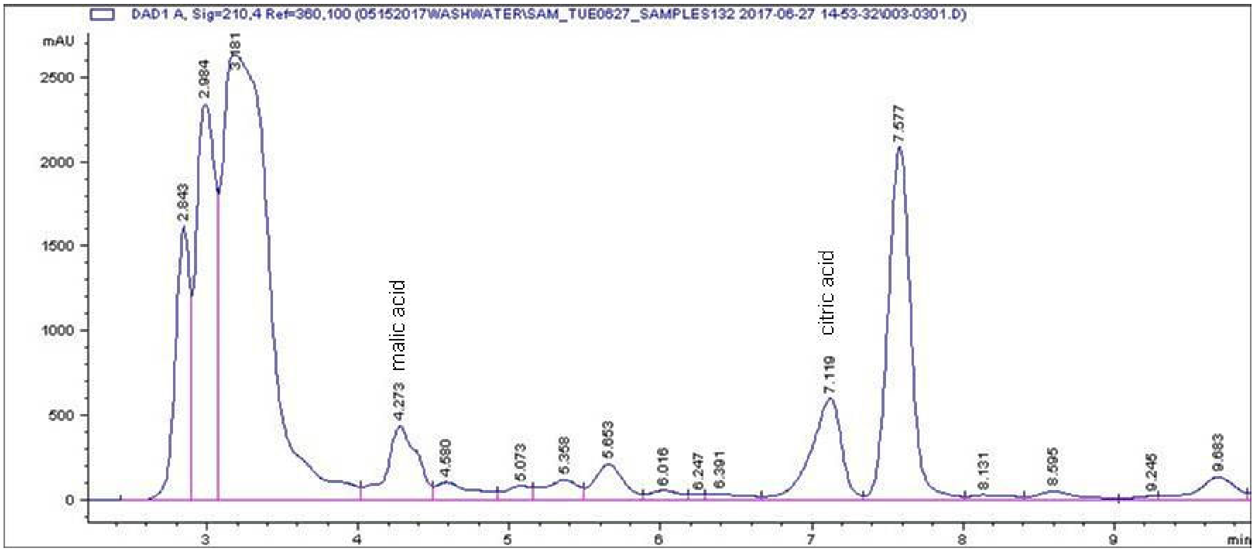

b)

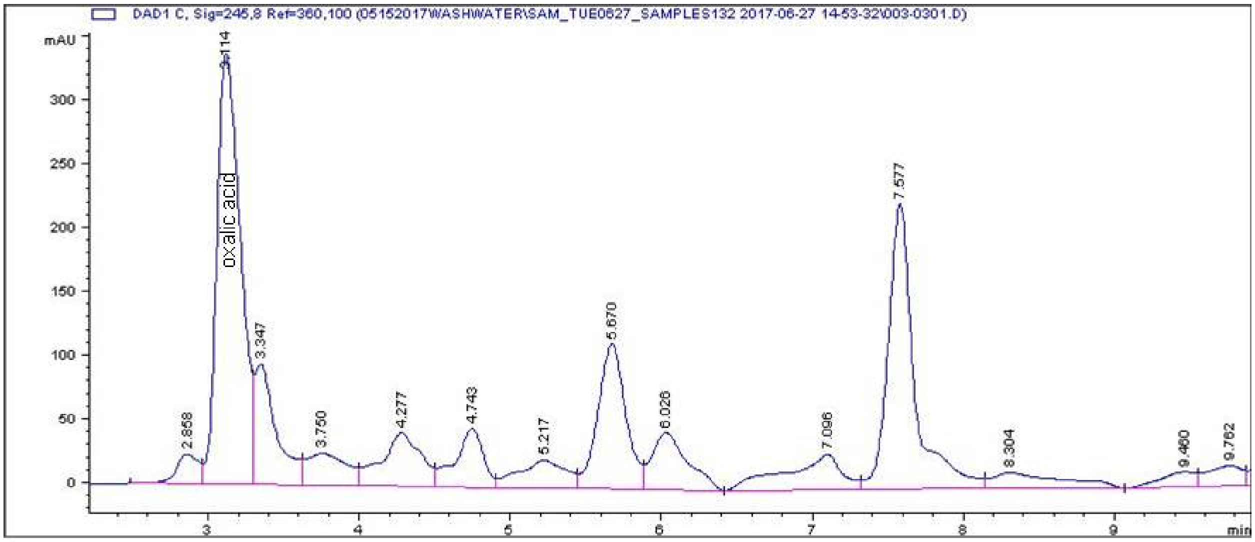

Supplement: S4 Fig — Representative chromatographs on cabbage wash water for a) absorbance at 210 nm (citric and malic acid), and b) absorbance at 246 nm (oxalic acid). (PDF) [file pone.0208945.s004.pdf]
