# Supplementary material for: Impacts and interactions of organic compounds with chlorine sanitizer in recirculated and reused produce processing water
Source: PLoS One. 2018 Dec 12;13(12):e0208945. doi: 10.1371/journal.pone.0208945 (PMC6291160; doi:10.1371/journal.pone.0208945)
Supplement: S1 Table — (PDF) [file pone.0208945.s005.pdf]

S1 Table. Composition of the high-MW fraction obtained in cabbage wash water

Compound	Abundance (mg/L)
Sugars	ND*
Proteins/peptides	163 ± 7
Total phenolics	ND
Acids (citric, malic, and oxalic)	ND

[*] Not detected.
