# Supplementary material for: Impacts and interactions of organic compounds with chlorine sanitizer in recirculated and reused produce processing water
Source: PLoS One. 2018 Dec 12;13(12):e0208945. doi: 10.1371/journal.pone.0208945 (PMC6291160; doi:10.1371/journal.pone.0208945)
Supplement: S2 Table — (PDF) [file pone.0208945.s006.pdf]

**S2 Table. Effect of chlorination on COD of cabbage wash water diluted to 1,500 mg/L COD**

| FC (mg/L) | COD (mg/L) † |
|---|---|
| 0 | 1,500[a] |
| 100 | 1,488 ± 12[ab] |
| 500 | 1,485 ± 7[ab] |
| 1000 | 1,477 ± 10[b] |

* Not detected.

† Different superscripts denote significant ($P < 0.05$) difference.
