# Supplementary material for: Impacts and interactions of organic compounds with chlorine sanitizer in recirculated and reused produce processing water
Source: PLoS One. 2018 Dec 12;13(12):e0208945. doi: 10.1371/journal.pone.0208945 (PMC6291160; doi:10.1371/journal.pone.0208945)
Supplement: S3 Table — (PDF) [file pone.0208945.s007.pdf]

**S3 Table. Polynomial models for predicting chlorine demand (y, in mg/L) of different compounds by their concentrations (x, in mg/L). The models are written as $y = ax^2 + bx + c$.**

| Compound | Range of x | y in 2 min | | | | y in 5 min | | | |
|---|---|---|---|---|---|---|---|---|---|
| | | a | b | c | $R^2$ | a | b | c | $R^2$ |
| Glucose | 1000~8000 | $-3 \times 10^{-7}$ | 0.004 | 3.214 | 0.954 | $3 \times 10^{-7}$ | $1 \times 10^{-4}$ | 8.112 | 0.999 |
| Fructose | 1000~8000 | $-1 \times 10^{-7}$ | 0.004 | 7.824 | 0.860 | $9 \times 10^{-9}$ | 0.002 | 24.11 | 0.985 |
| Citric acid | 50~400 | $-3 \times 10^{-4}$ | 0.307 | 28.72 | 0.999 | $-1 \times 10^{-4}$ | 0.202 | 45.62 | 0.994 |
| Malic acid | 100~500 | $-2 \times 10^{-4}$ | 0.272 | -5.844 | 0.998 | $2 \times 10^{-4}$ | 0.187 | 11.40 | 0.989 |
| Oxalic acid | 100~500 | $5 \times 10^{-5}$ | 0.053 | 13.11 | 0.992 | $5 \times 10^{-4}$ | -0.265 | 56.89 | 0.999 |
| SPH* | 10~200 | $-1 \times 10^{-3}$ | 1.513 | 8.246 | 0.999 | $-3 \times 10^{-3}$ | 2.138 | -1.482 | 0.984 |
| Gallic acid | 10~200 | $3 \times 10^{-4}$ | 2.177 | 6.045 | 0.999 | $-1 \times 10^{-3}$ | 2.726 | 0.606 | 0.999 |

| Compound | Range of x | y in 15 min | | | | y in 30 min | | | |
|---|---|---|---|---|---|---|---|---|---|
| | | a | b | c | $R^2$ | a | b | c | $R^2$ |
| Glucose | 1000~8000 | $-4 \times 10^{-7}$ | 0.008 | 3.662 | 0.960 | $-8 \times 10^{-7}$ | 0.018 | 0.904 | 0.993 |
| Fructose | 1000~8000 | $-1 \times 10^{-7}$ | 0.006 | 8.614 | 0.890 | $-1 \times 10^{-6}$ | 0.025 | 1.275 | 0.993 |
| Citric acid | 50~400 | $9 \times 10^{-4}$ | 0.194 | 71.35 | 0.904 | $2 \times 10^{-6}$ | 0.646 | 82.18 | 0.984 |
| Malic acid | 100~500 | $1 \times 10^{-4}$ | 0.931 | -18.13 | 0.972 | $6 \times 10^{-4}$ | 0.731 | 14.40 | 0.948 |
| Oxalic acid | 100~500 | $-3 \times 10^{-5}$ | 0.301 | -1.333 | 0.909 | $1 \times 10^{-5}$ | 0.100 | 97.58 | 0.997 |
| SPH* | 10~200 | $-3.1 \times 10^{-3}$ | 2.435 | 1.542 | 0.988 | $7 \times 10^{-4}$ | 1.685 | 8.068 | 0.999 |
| Gallic acid | 10~200 | $-1 \times 10^{-4}$ | 2.748 | 18.16 | 0.986 | $3 \times 10^{-4}$ | 2.766 | 28.50 | 0.997 |

| Compound | Range of x | y in 60 min | | | | y in 90 min | | | |
|---|---|---|---|---|---|---|---|---|---|
| | | a | b | c | $R^2$ | a | b | c | $R^2$ |
| Glucose | 1000~8000 | $1 \times 10^{-6}$ | 0.004 | 51.51 | 0.988 | $-5 \times 10^{-7}$ | 0.025 | 23.90 | 0.987 |
| Fructose | 1000~8000 | $2 \times 10^{-6}$ | 0.006 | 72.61 | 0.988 | $-7 \times 10^{-7}$ | 0.036 | 33.70 | 0.987 |
| Citric acid | 50~400 | $-9 \times 10^{-4}$ | 1.358 | 62.24 | 0.997 | $-6 \times 10^{-3}$ | 3.465 | 86.33 | 0.997 |
| Malic acid | 100~500 | $5 \times 10^{-4}$ | 0.403 | 68.68 | 0.998 | $4 \times 10^{-4}$ | 0.224 | 131.0 | 0.998 |
| Oxalic acid | 100~500 | $3 \times 10^{-4}$ | 0.026 | 101.9 | 0.956 | $-2 \times 10^{-4}$ | 0.444 | 79.89 | 0.989 |
| SPH* | 10~200 | $9 \times 10^{-5}$ | 1.880 | 28.70 | 0.999 | $1 \times 10^{-3}$ | 1.503 | 40.86 | 0.999 |
| Gallic acid | 10~200 | $3 \times 10^{-3}$ | 2.405 | 44.80 | 0.997 | $4 \times 10^{-3}$ | 2.132 | 61.96 | 0.999 |

| Compound | Range of x | y in 120 min | | | |
| --- | --- | --- | --- | --- | --- |
| | | a | b | c | $R^2$ |
| Glucose | 1000~8000 | -2 x $10^{-6}$ | 0.053 | 2.216 | 0.971 |
| Fructose | 1000~8000 | -2 x $10^{-6}$ | 0.058 | 2.438 | 0.971 |
| Citric acid | 50~400 | -2 x $10^{-4}$ | 1.881 | 66.00 | 0.912 |
| Malic acid | 100~500 | 1 x $10^{-4}$ | 0.686 | 22.17 | 0.962 |
| Oxalic acid | 100~500 | -6 x $10^{-4}$ | 0.740 | 39.20 | 0.971 |
| SPH* | 10~200 | 2 x $10^{-3}$ | 1.504 | 67.04 | 0.991 |
| Gallic acid | 10~200 | -2 x $10^{-3}$ | 2.498 | 60.20 | 0.993 |

* SPH: soy protein hydrolysate.
